# Processing of metacaspase into a cytoplasmic catalytic domain mediating cell death in *Leishmania major*

**DOI:** 10.1111/j.1365-2958.2010.07443.x

**Published:** 2011-01

**Authors:** Habib Zalila, Iveth J González, Amal Kuendig El-Fadili, Maria Belen Delgado, Chantal Desponds, Cédric Schaff, Nicolas Fasel

**Affiliations:** Department of Biochemistry, University of Lausanne155 Chemin des Boveresses, 1066 Epalinges, Switzerland

## Abstract

Metacaspases are cysteine peptidases that could play a role similar to caspases in the cell death programme of plants, fungi and protozoa. The human protozoan parasite *Leishmania major* expresses a single metacaspase (LmjMCA) harbouring a central domain with the catalytic dyad histidine and cysteine as found in caspases. In this study, we investigated the processing sites important for the maturation of LmjMCA catalytic domain, the cellular localization of LmjMCA polypeptides, and the functional role of the catalytic domain in the cell death pathway of *Leishmania* parasites. Although LmjMCA polypeptide precursor form harbours a functional mitochondrial localization signal (MLS), we determined that LmjMCA polypeptides are mainly localized in the cytoplasm. In stress conditions, LmjMCA precursor forms were extensively processed into soluble forms containing the catalytic domain. This domain was sufficient to enhance sensitivity of parasites to hydrogen peroxide by impairing the mitochondrion. These data provide experimental evidences of the importance of LmjMCA processing into an active catalytic domain and of its role in disrupting mitochondria, which could be relevant in the design of new drugs to fight leishmaniasis and likely other protozoan parasitic diseases.

## Introduction

Caspases are the main proteases activated during mammalian apoptosis mediating cleavage of a variety of proteins leading ultimately to cell death (Thornberry and Lazebnik, 1998; Los *et al*., 2001; Chowdhury *et al*., 2008; Li and Yuan, 2008). They have undergone remarkable evolution and specialization in vertebrates, in which they function in cell death cascades but also have been shown to be involved in development, inflammatory responses and lymphocyte proliferation (Lamkanfi *et al*., 2007).

In-depth comparative sequence and structural analysis of several eukaryote genomes revealed the presence of paracaspases and metacaspases, sharing the conserved catalytic histidine and cysteine dyad present in caspases, thus classifying them into the C14 clan CD cysteine protease family (Uren *et al*., 2000; Mottram *et al*., 2003). Paracaspases are expressed in mammalian cells and in some lower eukaryotes such as *Dictyostelium discoideum* (Roisin-Bouffay *et al*., 2004) whereas metacaspases are present in protozoa, fungi, plantae and chromista (Vercammen *et al*., 2007)*.* Substrate specificity of paracaspases and metacaspases is different from caspases since it is directed towards basic residues especially towards arginine in P1 position (Vercammen *et al*., 2004; 2006; Watanabe and Lam, 2005; González *et al*., 2007; Lee *et al*., 2007; Moss *et al*., 2007; Rebeaud *et al*., 2008). Finally, metacaspases were reported to be autocatalytically activated (Madeo *et al*., 2002; Watanabe and Lam, 2005; González *et al*., 2007) although, in the human protozoan parasite, *Trypanosoma brucei*, processing does not seem to be required for activity (Moss *et al*., 2007). In unicellular organisms, the benefit of programmed cell death (PCD) is less evident than in mammals. There are, however, increasing experimental evidence that unicellular organisms undergo PCD under certain conditions. In protozoan parasites, cell death was described in *Plasmodium* species, in *Trypanosoma* and in *Leishmania* (Deponte, 2008).

*Leishmania* parasites are transmitted to humans by the bite of a sand fly insect vector. In the insect stage, the procyclic promastigotes proliferate as free-living flagellated forms within the midgut where they then differentiate into virulent metacyclics, and are then able to migrate to the proboscis (da Silva and Sacks, 1987). Once in the mammalian host, promastigotes are phagocytosed by macrophages, and transformed into round non-flagellated intracellular forms, the amastigotes, causing leishmaniasis in the host. In the presence of nitric oxide (NO) or reactive oxygen species (ROS), as produced by the innate immune response of the host, or anti-*Leishmania* drugs, morphological and biochemical features of cell death have been reported in different *Leishmania* species (Moreira *et al*., 1996; Das *et al*., 2001; 2008; Sereno *et al*., 2001; Arnoult *et al*., 2002; Holzmuller *et al*., 2002; Lee *et al*., 2002; Mukherjee *et al*., 2002; Zangger *et al*., 2002; Marquis *et al*., 2003; Nguewa *et al*., 2004; Paris *et al*., 2004; Verma and Dey, 2004; Alzate *et al*., 2006; 2007; Sen *et al*., 2007a,b; Casanova *et al*., 2008). This includes increase in intracellular calcium, mitochondrial depolarization, release of cytochrome *c* and apoptosis inducing factor, phosphatidylserine (PS) exposure, plasma membrane permeability, cell cycle arrest, ROS generation, translocation of endonuclease G, chromatin condensation and DNA laddering.

A cell death programme in *Leishmania* could be essential in controlling the density of the parasite population in the insect stage or in the host macrophage. Lately, it was shown that dead parasites present in the inoculum are important for the virulence by modulating the innate immune response (van Zandbergen *et al*., 2006; Wanderley *et al*., 2009). However, thus far, the pathway leading to cell death is mainly unknown, although, in some reports, caspase-like activity has been associated with PCD in *Leishmania* species. Metacaspase, with its caspase-like catalytic domain, could be one of the cell death executioner proteases fulfilling the functional role of mammalian caspases in PCD pathways. However, because of its substrate specificity, the metacaspase cannot be accounted for detection of caspase-like activity and therefore the cell death proteolytic degradation pathway in *Leishmania* is likely to be different.

In *Leishmania major*, a single gene codes for a metacaspase polypeptide of 435 amino acids (LmjMCA). Analysis of the amino acid sequence shows that LmjMCA can be divided into three main domains: an N-terminus sequence which contains a mitochondrial localization sequence, a catalytic domain with the histidine/cysteine dyad and a C-terminus segment rich in proline (Ambit *et al*., 2008). Based on these structural features, it would be reasonable to hypothesize that LmjMCA is targeted to the mitochondrion and that LmjMCA is active in such compartment. Functional heterologous complementation of a *Saccharomyces cerevisae* metacaspase null mutant by *LmjMCA* showed that LmjMCA is autocatalytically cleaved and could be one of the executioner proteases of the H_2_O_2_-induced PCD (González *et al*., 2007). Moreover, we have demonstrated that, in yeast, the processing of *Leishmania* metacaspase and the release of an active domain were responsible of the increase of its activity towards arginine substrates (González *et al*., 2007) suggesting that LmjMCA processing to enzymatically active protein could be involved in PCD. Similarly, it was shown that full-length *Leishmania donovani* metacaspase, when overexpressed, participated in the cell death pathway (Lee *et al*., 2007). Thus, although *Leishmania* MCA could have several functions, it is likely that one of them is related to cell death. However, this latter function has not been related yet to the LmjMCA processing and to enzymatic activity of the catalytic domain in oxidative stress situations or in the presence of anti-*Leishmania* drugs.

The possible role of LmjMCA in cell death raises the questions whether LmjMCA is matured into a defined catalytic domain in oxidative stress or in the presence of leishmanicidal drugs and whether this domain is sufficient for LmjMCA function in cell death. Considering that the catalytic domain of LmjMCA could be localized in different cellular compartments due to the presence of a mitochondrial localization signal (MLS) on the primary translation product and that this precursor could be processed into several polypeptides, it was therefore important to define the processing sites delimiting the catalytic domain, to determine its cellular localization in order to understand the origin of a possible death signal and finally whether overexpressing LmjMCA catalytic domain (LmjMCA-cd) would enhance susceptibility of parasites to stress.

## Results

### Metacaspase catalytic domain localization

**Fig. 1:**
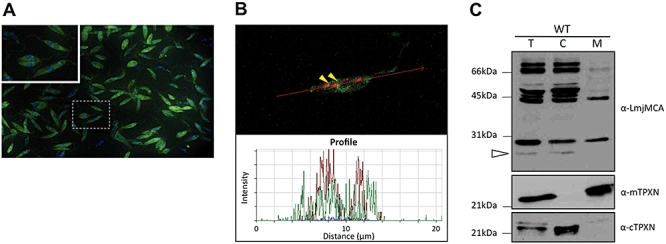
LmjMCA intracellular localization. A. Endogenous LmjMCA localization was analysed by immunofluorescence using an anti-LmjMCA antibody (green). Nuclei and kinetoplasts were stained with DAPI (blue). B. Confocal microscopy of LmjMCA-overexpressing parasites. Mitochondrion was stained with Mitotracker (red) and a signal/distance profile was generated in order to confirm the colocalization. C. *L. major* parasites subcellular fractionation was obtained by digitonin treatment. T (total) corresponds to total proteins, C (cytoplasm) corresponds to 100 µM digitonin and M (mitochondria) corresponds to 500 µM digitonin. Proteins were immunoblotted using RE53. Anti-mTPXN (mitochondrial tryparedoxin peroxydase) anti-cTPXN (cytoplasmic tryparedoxin peroxydase) were used as fractionation controls.

LmjMCA secondary structure is characterized by the presence of a possible N-terminal MLS (AA 1–29) suggesting that LmjMCA and its catalytic domain could be targeted to the single mitochondrion present in trypanosomatids. As a preliminary experiment to determine the cellular localization of LmjMCA and its catalytic domain, we analysed the endogenous expression of LmjMCA by immunofluorescence using the RE53 antibody recognizing the LmjMCA-cd (González *et al*., 2007). Nuclei and kinetoplasts were stained with DAPI. LmjMCA-cd localization was likely to be cytoplasmic (Fig. 1A). To gain in resolution and in detection sensitivity, we performed confocal microscopy, stained the mitochondrion with red Mitotracker and used a signal/distance profile to evaluate any colocalization with an overexpressed C-terminally tagged LmjMCA (Fig. 1B). Although we could detect a partial colocalization of LmjMCA with the mitochondrion (arrowheads in Fig. 1B) the confocal image showed mainly a cytosolic localization. With the signal/distance profile analysis, we could confirm that most of the LmjMCA was localized in the cytoplasm, although, albeit at low level, some LmjMCA could be present in the mitochondrion, as evidenced by the overlapping red and green signal/distance profiles in Fig. 1B.

**Fig. 2:**
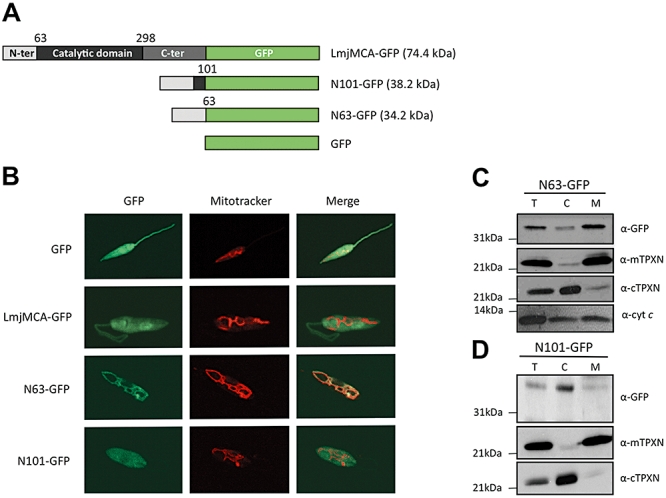
Localization of LmjMCA–GFP fusion proteins. A. Schematic representation of the LmjMCA–GFP fusion proteins obtained after insertion of the corresponding sequences in the pNUS–GFPcN expression vector. LmjMCA–GFP corresponds to the full-length LmjMCA tagged with GFP; N101–GFP and N63–GFP correspond to the 101 and 63 LmjMCA first amino acids fused to GFP; GFP corresponds to the GFP protein expressed by the pNUS–GFPcN vector. B. Parasites expressing GFP, LmjMCA–GFP, N63–GFP and N101–GFP were analysed after Mitotracker staining and observed by confocal microscopy. C and D. Subcellular fractions were obtained by digitonin treatment. T (total) corresponds to total proteins, C (cytoplasm) corresponds to 100 µM digitonin and M (mitochondria) corresponds to 500 µM digitonin. Proteins from N63–GFP (C) and N101–GFP (D) parasites were immunoblotted with a monoclonal anti-GFP antibody. Anti-mTPXN (mitochondrial tryparedoxin peroxydase), anti-cTPXN (cytoplasmic tryparedoxin peroxydase) and anti-cyt *c* (cytochrome *c*) were used as fractionation controls.

**Fig. 4:**
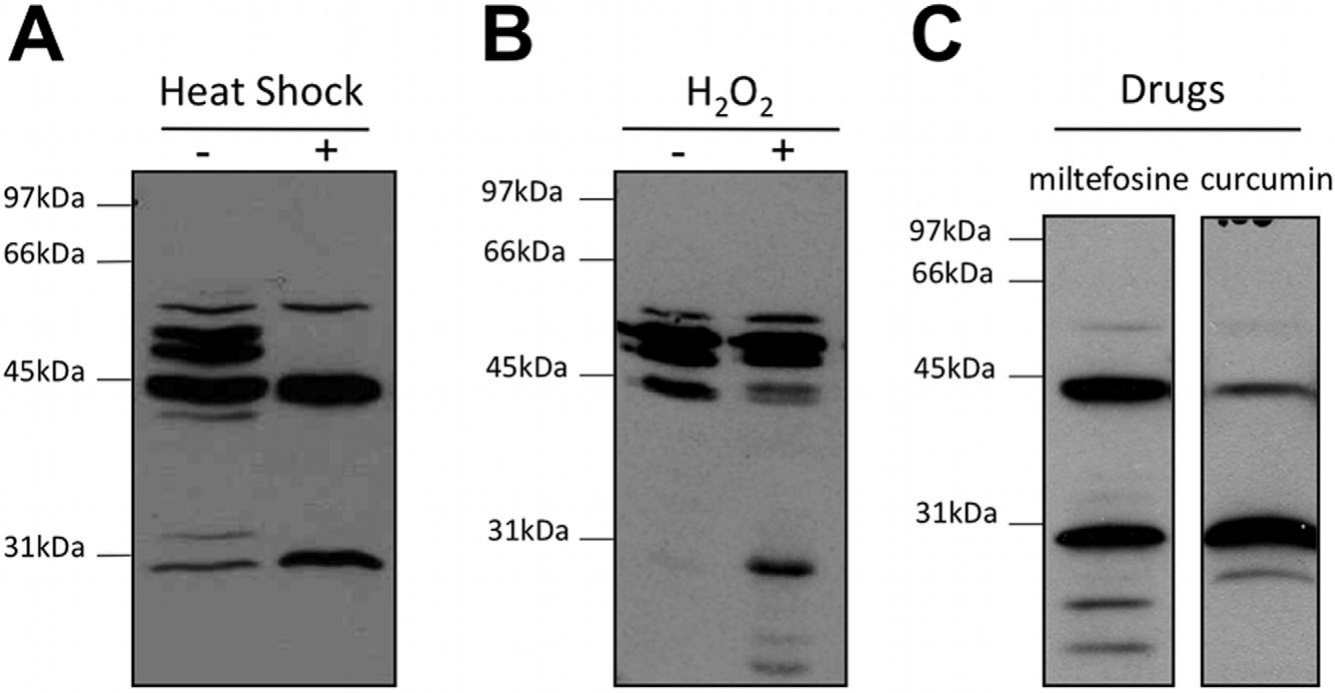
LmjMCA processing during cell death. A. *L. major* promastigotes were heat shocked during 10 min at 55°C then incubated during 1 h at 26°C. NP-40-soluble proteins were immunoblotted using RE53. (−): non-treated; (+): treated. B. *L. major* promastigotes were treated with 1 mM hydrogen peroxide during 1 h and NP-40-soluble proteins were analysed by immunoblot with RE53. (−): non-treated; (+): treated. C. *L. major* promastigotes were treated during 10 h with 40 µM miltefosine or 100 µM curcumin and NP-40-soluble proteins were analysed by immunoblot using RE53.

To investigate biochemically the cellular localization of LmjMCA polypeptides, we used digitonin fractionation to separate mitochondrial and cytoplasmic proteins (Foucher *et al*., 2006). Logarithmic-phase parasites were lysed using different concentrations of digitonin and proteins representing cytoplasmic or mitochondrial fractions were separated by SDS-PAGE and analysed by immunodetection using the RE53 antibody (Fig. 1C). In parallel, as control, total proteins were analysed using RE53 antibody. Simultaneously, the quality of our fractionation procedure was estimated using polypeptides having specific localizations, mitochondrial and cytoplasmic TPXN (mTPXN and cTPXN respectively). As shown, using specific antibodies, mTPXN is present in the mitochondrial fraction whereas cTPXN is present only in the cytoplasm as expected (Castro *et al*., 2002). After lysis with 100 µM digitonin, to enrich for cytoplasmic proteins, the cytoplasmic fraction was analysed using RE53. In addition to the detection of cross-reacting polypeptides which migrate above the 66 kDa molecular weight marker and therefore could not correspond to LmjMCA (González *et al*., 2007) and are detected only when digitonin is used (refer to Fig. 4), the pattern of LmjMCA polypeptides detected in the cytoplasmic fraction (fraction C) was similar to the pattern of LmjMCA polypeptides detected in total lysates of *L. major* parasites (fraction T). Three main bands were detected in the 45 kDa range, the upper band could correspond to the full-length LmjMCA protein (47.2 kDa) and the two lower bands should be partially processed catalytic domain-containing precursors. Two additional bands were recognized by the anti-catalytic domain antibody: one major processed product which was present in the cytoplasmic (C) and mitochondrial (M) fractions and migrating with an apparent molecular mass of 28 kDa and a minor product which migrated with an apparent molecular mass of 25 kDa. When the mitochondrial fraction was analysed (M), most of the higher molecular weight LmjMCA polypeptides were not detected by RE53. Only processed forms could be detected. In the lower molecular weight range, the processed product with an apparent molecular mass of 28 kDa was detected in the C and the M fractions, whereas the minor product appeared to be restricted to the cytoplasmic fraction (shown by an arrowhead). These data showing a dual repartition of LmjMCA suggest that a LmjMCA protein subset would be targeted into the mitochondrion where it undergoes processing and some forms are only present in the cytoplasm. Thus, LmjMCA could be distributed between two different compartments. Based on the presence of mTPXN in the 500 µM digitonin fraction and knowing that mTPXN is localized in the matrix and that digitonin at 100 µM concentration solubilized the mitochondrial outer membrane as evidenced by the partial release of cytochrome *c* (Fig. 2C, lower panel), we can also conclude that processed forms of LmjMCA are localized in the mitochondrial matrix and not in the intermembrane space.

To further evaluate the functionality of LmjMCA MLS and to obtain additional information on the cellular localization, on the regulated targeting process of LmjMCA polypeptides and on the processing of LmjMCA in the mitochondrion, GFP was fused at the C-terminus of the first 63 (N63–GFP) or 101 amino acids (N101–GFP) of LmjMCA (Fig. 2A). Both constructs encode the MLS sequence and could be thus expected to translocate GFP into the mitochondrion. Transfected parasites expressing either constructs or the *GFP* gene were analysed by confocal microscopy using Mitotracker to evaluate localization of LmjMCA in the mitochondrion. When GFP is expressed in *Leishmania* parasites, fluorescence is only visible in the cytoplasm (Fig. 2B). On the contrary, the N63–GFP polypeptide was targeted to the mitochondrion as we can see by its colocalization with the Mitotracker staining (Fig. 2B) confirming that LmjMCA possesses a functional MLS. The mitochondrial targeting was observed neither for the N101–GFP nor for the full-length LmjMCA–GFP suggesting that these polypeptides and their processed products are mainly retained in the cytoplasm.

The cellular compartmentalization of both GFP-tagged LmjMCA constructs (N63–GFP and N101–GFP) was further analysed biochemically using digitonin fractionation and an anti-GFP monoclonal antibody. Similarly to the previous experiment, quality of the fractionation procedure was verified using antibodies recognizing specifically cTPXN or mTPXN.

As expected for an efficient targeting to the mitochondrion and consistent with the microscopic localization, N63–GFP protein was found to localize in the mitochondrial fraction (Fig. 2C). As these proteins were also present at low level in the cytoplasmic fraction (fraction C), we have then added an additional control for the mitochondrial inter-membrane space (cytochrome *c*) and found that digitonin solubilized the mitochondrial outer membrane and thus provoked a partial release of proteins present in the mitochondrial inter-membrane space into the cytoplasm. We can thus consider that the M fraction is enriched in mitochondrial matrix proteins or proteins that are associated with the inner membrane and that N63–GFP is efficiently targeted into the mitochondrion matrix or associated with the inner membrane. Based on the presence of mTPXN in the 100 µM digitonin fraction and knowing that this tryparedoxin peroxidase is localized in the mitochondrial matrix, we can conclude that N63–GFP is also localized in the mitochondrial matrix and not in the intermembrane space. We can exclude processing of N63–GFP in the cytoplasm, since fluorescence of N63–GFP was observed only in the mitochondrion and not in the cytoplasm (Fig. 2B). Interestingly, in contrast to the N63–GFP, N101–GFP was mainly cytoplasmic and was not detected in the mitochondrion albeit the presence of MLS at its N-terminus (Fig. 2B). This result suggests that the AA sequence 64–101 could play a role in preventing most of the translocation of N101–GFP and consequently translocation of LmjMCA into the mitochondrion. This microscopic observation was also validated biochemically by digitonin cell fractionation (Fig. 2D). Structurally, in comparison with the N63–GFP, the N101–GFP contains several hydrophobic residues between amino acids 64 and 101. Further investigations are needed to determine whether and how these amino acids could limit the targeting to the mitochondrion.

In any case, our microscopy and biochemical fractionation experiments allowed us to conclude that, although a functional MLS is present at the N-terminus of LmjMCA, most (but not all) of the catalytic domain-containing molecules are detected in the cytoplasm and not translocated into the mitochondrion.

### Processing of LmjMCA and maturation of LmjMCA catalytic domain

**Fig. 3:**
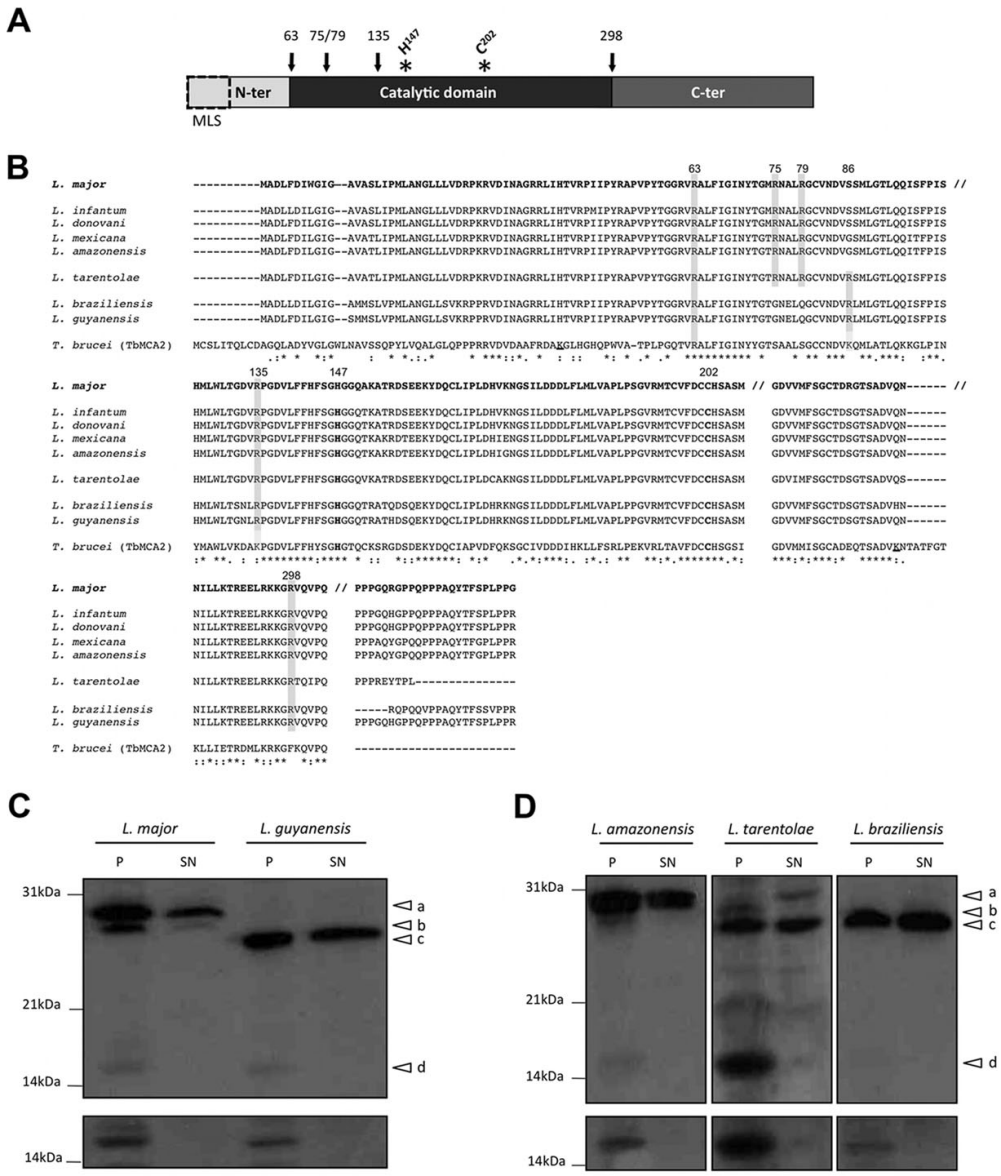
*Leishmania* metacaspase sequence comparison. A. Schematic representation of LmjMCA sequence showing a predicted N-terminal (N-ter) domain (light grey) with the mitochondrial localization signal (MLS) from amino acid 1 to 29, a caspase-like catalytic domain (black) with the catalytic dyad H147 and C202, and a C-terminal (C-ter) proline-rich extension (dark grey). Arrows denote the possible cleavage sites at positions 63, 75/79, 135 and 298. B. *L. major* (Uniprot Accession No.: Q4FWW2), *L. infantum* (A4IB59), *L. donovani* (Q0ZL29), *L. mexicana* (B6DU81), *L. amazonensis* (B6DU80), *L. tarentolae* (B6DU79), *L. braziliensis* (B6DU82), *L. guyanensis* (B6DU83) and *T. brucei* (TbMCA2, Q585F3) metacaspase amino acid sequences were aligned using clustal w2 program. Arginines in P1 position of potential cleavage sites are highlighted. The catalytic dyad, H147 and C202, is shown in bold and lysine cleavage sites of TbMCA2 (K55 and K268) are underlined. C and D. Comparison of metacaspase processing profile between *Leishmania Leishmania* (*L. major, L. amazonensis*), *Sauro-Leishmania* (*L. tarentolae*) and *L.* (*Viannia*) subgenus (*L. guyanensis, L. braziliensis*) species. Insoluble (P) and soluble (SN) proteins were immunoblotted using RE53. Arrowheads indicate the catalytic domain-containing processed forms of metacaspase. a: R63–298 LmjMCA fragment; b: R75/79–298 LmjMCA fragment; c: R86–298 LmjMCA fragment; and d: R135–298 LmjMCA fragment. The lower panels show the same autoradiograms at longer exposure.

LmjMCA has a molecular mass of 47.2 kDa and is encoded by a single gene (*LmjF 35.1580*). Its secondary structure is characterized by the presence of a possible N-terminal MLS (AA 1–29), a predicted caspase-like domain (AA 63–314) (Sanger Pfam database; Accession No. Q4FWW2_LEIMA) with the conserved catalytic dyad His147/Cys202, and a C-terminal proline-rich domain (amino acid residues 315–435) (Fig. 3A). When LmjMCA expression and processing are analysed in parasites, several polypeptides are specifically detected by a rabbit antibody recognizing LmjMCA-cd (González *et al*., 2007). These polypeptides are likely to correspond to the 47.2 kDa primary translation product of *LmjMCA* gene and different processed forms of lower molecular masses. This observation suggested that LmjMCA precursor is not fully processed, is cleaved at different positions, and that the catalytic domain is present in several of these polypeptides. Based on the size of the predicted caspase-like domain (63–314), on its preferential specificity towards substrates harbouring mainly arginine in the P1 position and finally on the functional complementation of a *S. cerevisae* metacaspase null mutant by a polypeptide encoding only the *LmjMCA* caspase-like domain (AA 63–314) (González *et al*., 2007), we can hypothesize that LmjMCA cleavage sites should be conserved in other *Leishmania* species. Therefore, to define the limits of LmjMCA catalytic domain, we compared the primary amino acid sequences of MCA from several *Leishmania*, *Leishmania* (*Viannia*) and *Sauro-Leishmania* subgenus species (Fig. 3B). As shown in this figure, the general structure is conserved between the different species. To generate a mature catalytic domain in the range of the processed products detected in Fig. 1C and migrating with molecular masses lower than the 31 kDa marker, cleavage at the N-terminus could occur at positions of arginine 63 (R63), arginine 75 and/or 79 (R75/79) in every species except in *L.* (*Viannia*) species. In this latter subgenus, cleavage at R75/79 could be replaced by a processing at position of arginine 86 (R86). If, in this subgenus, R86 is used as a cleavage site at the N-terminus, and at position of arginine 298 (R298) on the C-terminus side, as predicted from the MCA primary amino acid sequence in every *Leishmania* species, the catalytic domain released after cleavage should be of lower molecular mass in the *L.* (*Viannia*) subgenus. Furthermore, according to the amino acid sequence, cleavage at R63–298, R75/79–298 and R86–298 should generate polypeptides in the ranges of 26, 24 and 23 kDa respectively. *Leishmania* metacaspase sequences were also aligned to *T. brucei* metacaspase TbMCA2, which shows the highest degree of homology to *Leishmania* metacaspase. TbMCA2 does not seem to be processed at arginine since autoprocessing of TbMCA2 was reported to occur at lysine K55 and K268 (Moss *et al*., 2007) although TbMCA2 exhibited preferential cleavage especially towards substrates exhibiting arginine in P1 position (Moss *et al*., 2007). The reasons for such discrepancies between *Leishmania* and *T. brucei* are unknown. Further investigations are required to solve this issue.

To verify experimentally these hypotheses, logarithmic-phase *Leishmania* parasites of different species were lysed in the presence of NP-40 and proteins were analysed using a rabbit antibody (RE53) recognizing the catalytic domain of LmjMCA (González *et al*., 2007). In *L. major*, two polypeptides in the low molecular weight range are detected in both soluble and insoluble fractions. Both have molecular masses larger than the *in silico* predicted molecular mass likely due to the amino acid composition affecting the migration in SDS polyacyamide gels. Biochemically, based on the difference in migration, they could represent two cleavage events, namely at R63–298 and R75/79–298. Thus, the difference in migration between these two polypeptides can be explained by a differential processing. As shown in Fig. 3C, a difference in the migration is observed between *L. major* and *L. guyanensis*. As predicted from the primary amino acid sequence analysis, a single band with a molecular mass lower than the one in *L. major* is detected in *L. guyanensis*. These data strongly suggest that cleavage at the C-terminus (R298) is conserved in different *Leishmania* species but that cleavage at the N-terminus occurs at different positions in *L. major* and in *L. guyanensis*, e.g. at R86 in *L. guyanensis* and at R63 and R75/79 in *L. major*. This observation was validated by analysing other *Leishmania Leishmania* and *L.* (*Viannia*) subgenera (Fig. 3D). *Leishmania amazonensis* showed a R63–298 band similar to *L. major* and *L. braziliensis* a R86–298 band similarly to *L. guyanensis*. Interestingly, the lizard parasite, *Leishmania tarentolae*, belonging to the *Sauro-Leishmania* subgenus, whose amino acids sequence harbours the R63, R75/79 and R86 sites, showed the R63–298, R75/79–298 and R86–298 bands.

Based on amino acid comparison, it is conceivable that the cleavage at arginine 135 (R135) which is conserved in every species is used also in the maturation of LmjMCA. In such a case, as predicted by the amino acid sequence, migration should be similar in every species and a polypeptide with a molecular mass of 17.6 kDa should be detected. As expected for processing at R135 and R298, a polypeptide of 18 kDa was detected in the NP-40-insoluble fraction of each parasite species analysed using RE53. It should be mentioned that this polypeptide is likely to be present at a low level since it is visible only on longer exposure in all species except *L. tarentolae* where it appears to be constitutively highly expressed (Fig. 3C and D, lower panels).

To confirm cleavage of LmjMCA at position R79, we expressed a LmjMCA-Flag polypeptide construct and compared its processing with a mutated form harbouring alanine instead of arginine at position 79. As shown in Fig. S1A, the 39 kDa polypeptide species detected in the wild-type form is not detected when arginine 79 was changed to alanine confirming our hypothesis (Fig. S1A, arrowhead a). To confirm that cleavage can occur at R298, we had to change both R63 and R298 to alanine to circumvent interdependent cleavage events. As shown in Fig. S1B, we detected a 42 kDa product using an anti-GFP antibody. This polypeptide corresponding to a full-length LmjMCA–GFP polypeptide processed at R298 is not detected in parasites expressing the LmjMCA–GFP R63A/R298A construct (Fig. S1B, arrowhead b). Interestingly, the 34 kDa band detected by the anti-GFP is also affected when the processing at R63 and R298 is blocked (Fig. S1B, arrowhead c). Thus far, we were not able to map this processing site, which is in any case downstream of the catalytic domain.

### Processing of LmjMCA in the cell death pathway

In physiological conditions, LmjMCA is processed in two sets of polypeptides consisting of high-molecular-weight precursors in the 45 kDa range and low-molecular-weight processed forms in the 25–30 kDa range. These latter polypeptides were expressed at different levels and present in the NP-40-insoluble fraction. According to the expression of LmjMCA-cd in yeast, it was likely that lower-molecular-weight polypeptides correspond to the catalytic domain derived by processing of the precursor forms and such polypeptides could be important in *Leishmania* cell death pathway (González *et al*., 2007). However, it was impossible to conclude whether any of these catalytic domain-containing polypeptides are important for cell death in parasites. Assuming that, like for caspases, LmjMCA should be activated by processing and maturation of the catalytic domain, we analysed the expression and processing of LmjMCA in parasites exposed to different stress conditions. We first analysed the expression and processing of LmjMCA when cell death was induced by heat shock (HS) at 55°C for 10 min followed by an incubation period of 1 h at 26°C, then lysed by NP-40 and soluble fractions were analysed by immunoblotting using the rabbit antiserum RE53 recognizing the catalytic domain of LmjMCA. In comparison with non-treated parasites exhibiting several LmjMCA polypeptides (Fig. 4A), two main protein species are detected in the treated sample: a band migrating with a molecular mass of 45 kDa and a band with a molecular mass of 26 kDa likely corresponding to the R63–298 catalytic domain. Interestingly, these two LmjMCA processed forms were found to be present in the cytoplasmic and in the mitochondrial fractions under physiological conditions (Fig. 1C).

Expression and processing of LmjMCA were also investigated when PCD was induced by oxidative stress. Protein fractions of *L. major* promastigotes treated with 1 mM hydrogen peroxide (H_2_O_2_) at 26°C for 1 h were analysed by immunoblot with anti-LmjMCA antibodies (Fig. 4B). Although, in the conditions used, LmjMCA did not show extensive degradation, a processed product migrating with a molecular mass of 26 kDa was detected by the anti-catalytic domain antibody, similarly to the 26 kDa product detected when parasites were treated by heat shock.

To determine whether such a pattern is also present when parasites are exposed to drugs, *Leishmania* promastigotes were grown in the presence of miltefosine or curcumin. Analysis of NP-40-soluble protein fractions from parasites undergoing drug-induced PCD using leishmanicidal drugs such as miltefosine (Paris *et al*., 2004; Verma and Dey, 2004) or curcumin (Das *et al*., 2008) showed the same processing pattern of LmjMCA as in HS and H_2_O_2_-treated parasites, with extensive cleavage of precursor forms and accumulation of low-molecular-weight products (Fig. 4C). Although some minor degradation products are detected and could be due to the extensive proteolytic degradation occurring in dying parasites, we can conclude from these data that the accumulation of catalytic domain-containing forms due to processing of LmjMCA precursors could be a feature common to oxidative stress and drug-induced cell death in *Leishmania* parasites.

### Overexpression of LmjMCA enhanced sensitivity of *Leishmania* parasites to H_2_O_2_

**Fig. 5:**
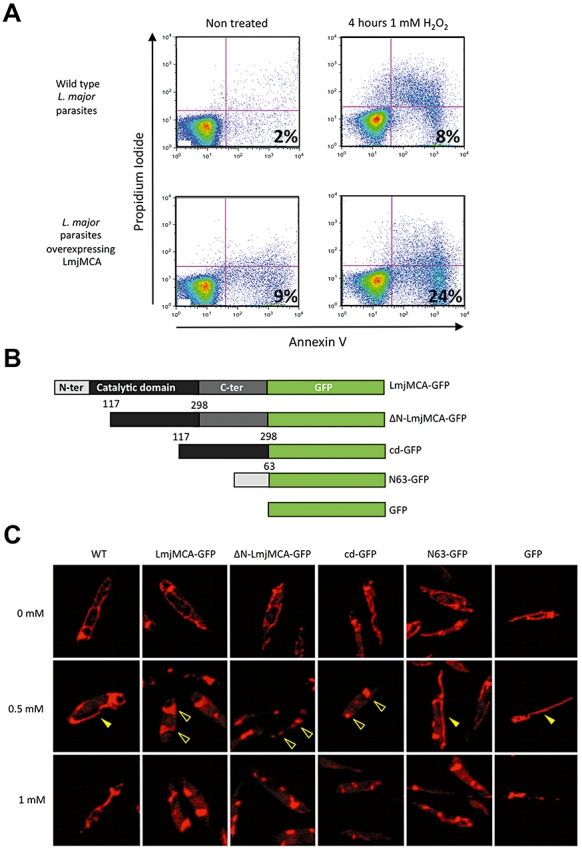
LmjMCA effect on H_2_O_2_-induced programmed cell death. A. Phosphatidylserine (PS) exposure at the membrane of *L. major* promastigotes treated with H_2_O_2_. Wild-type parasites and parasites overexpressing LmjMCA were not treated (left panels) or treated (right panels) with 1 mM H_2_O_2_ for 4 h. Cells were stained with fluorescein isothiocyanate (FITC)-labelled annexin V and PI to distinguish between apoptotic (annexin V-positive, PI-negative) and necrotic (PI-positive, annexin V-negative) cells. B. Schematic representation of the LmjMCA–GFP fusion proteins. LmjMCA–GFP corresponds to the full-length LmjMCA tagged with GFP; ΔN-LmjMCA–GFP corresponds to a GFP-tagged metacaspase without the N-terminus, cd–GFP corresponds to the catalytic domain of LmjMCA tagged with GFP, N63–GFP corresponds to the 63 LmjMCA first amino acids fused to GFP and GFP corresponds to the GFP protein expressed from the cloning vector. C. The generated fusion proteins were expressed in *L. major* parasites and mitochondrial function of H_2_O_2_-treated (0.5 or 1 mM) logarithmic-phase promastigotes was monitored by confocal microscopy after Mitotracker staining. Wild-type parasites were compared with parasites overexpressing the metacaspase (LmjMCA–GFP), the metacaspase without the N-terminus (ΔN-LmjMCA–GFP), the catalytic domain (cd–GFP) or the N-terminal domain alone (N63–GFP).

**Fig. 6:**
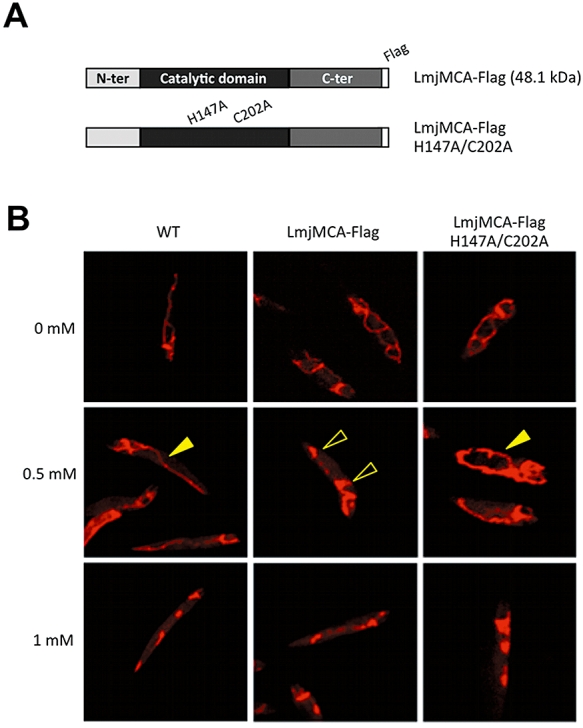
Catalytic activity-dependant effect LmjMCA. A. Schematic representation of the C-terminally M2 Flag-tagged LmjMCA protein (LmjMCA-Flag) and its enzymatically inactivated form (LmjMCA-Flag H147A/C202A). B. Generated fusion proteins were expressed in *L. major* parasites and analysed as in Fig. 5C. Solid arrowheads indicate intact mitochondria and open arrowheads indicate affected mitochondria.

Similarly to overexpression of executioner proteases (e.g. caspases) or nucleases (e.g. endonuclease G) in mammalian cells (Ryter *et al*., 2007), overexpression of *L. donovani* MCA (Lee *et al*., 2007) or endonuclease G (Gannavaram *et al*., 2008; Rico *et al*., 2009) enhanced sensitivity of parasites to oxidative stress. Furthermore, expression of LmjMCA catalytic domain in a *S. cerevisae* metacaspase null mutant (*Δyca-1*) complemented the cell death function of *YCA-1* (González *et al*., 2007). These data suggest that overexpression of cell death executioners is one approach to define the role of proteins in a cell death pathway and that LmjMCA-cd could play an important role in the *Leishmania* cell death pathway. First, we confirmed that overexpression of LmjMCA induced phenotypic markers of cell death. To this end, *L. major* parasites were transfected with a construct expressing LmjMCA and phosphatidylserine exposure at the cell surface was measured using annexin V in comparison with non-transfected parasites. Propidium iodide (PI) was used simultaneously to estimate the percentage of cells presenting a necrotic phenotype. As shown in one representative experiment (Fig. 5A), in normal culture conditions, when cells were analysed by FACS, non-transfected parasites showed 2% of annexin V positive (AnV+) cells. When these cells were incubated for 4 h in 1 mM H_2_O_2_, the percentage of AnV+ cells raised to 8%. The percentage of double positive cells (PI and AnV) increased from 0.7% to over 10% when H_2_O_2_ was present. In LmjMCA-overexpressing parasites, the percentage of AnV+ parasites raised from 9% to over 24% in the presence of H_2_O_2_ and the increase in double positive was less marked than in non-transfected cells. This result confirmed that LmjMCA overexpression could play a role in cell death and suggest that it could control the pathway and limit necrosis. We decided therefore to investigate the different parasite lines we used in this study for their potential effect on *Leishmania* death in the presence of H_2_O_2_. Untransfected wild-type *L. major* parasites and parasites transfected with LmjMCA–GFP, LmjMCA–GFP lacking the N-terminal domain, the catalytic domain of LmjMCA fused to GFP and finally N63–GFP (Fig. 5B) were analysed for their function in the presence of increasing concentrations of H_2_O_2_. Parasites were incubated with Mitotracker then exposed to two different concentrations of H_2_O_2_. In non-transfected cells or parasites expressing either N63–GFP or the GFP, incubation in the presence of 0.5 mM H_2_O_2_ had no effect on the mitochondrion (Fig. 5C, solid arrowheads) and at least 70% of the parasites exhibited intact mitochondrion as quantified in Table 1. In parasites overexpressing either a full-length LmjMCA, an N-terminus truncated LmjMCA, ΔN-LmjMCA–GFP or the catalytic domain, cd–GFP, a concentration of 0.5 mM H_2_O_2_ provoked an obvious mitochondrial loss of function (Fig. 5C, open arrowheads) as evidenced by the loss of Mitotracker fluorescence with less than 3% of the parasites still having intact mitochondrion (Table 1). When the concentration of H_2_O_2_ was raised to 1 mM, all the lines started to exhibit the same phenotype at the mitochondrial level. The level of expression of GFP-tagged proteins was analysed by immunoblot using an anti-GFP antibody. We detected GFP-fused polypeptides at relatively similar levels and can therefore exclude that the different phenotypes are accounted for by differences in the level of expression (data not shown). These data provide for the first time evidence that LmjMCA and in particular its catalytic domain plays a role in *Leishmania* cell death pathway, likely by acting on the mitochondrion, enhancing its susceptibility to oxidative stress. Moreover, as observed when the catalytic domain alone was expressed, there was no need for this domain to be targeted into the mitochondrion to exert its effect. To verify that the effect visible on the mitochondrion function was due to the enzymatic activity of LmjMCA and not to any other function of the catalytic domain, we performed a similar experiment with an inactive LmjMCA (Fig. 6A). As expected, mutating the catalytic dyad into alanines reverted to mitochondrial loss of function phenotype observed at 0.5 mM H_2_O_2_ in LmjMCA-overexpressing parasites (arrowheads in Fig. 6B and Table 1). As previously, we excluded that the different phenotypes are accounted for by differences in the level of expression (data not shown).

**Table 1:** Percentage of cells with intact mitochondrion after 1 h H_2_O_2_ treatment.

	0 mM	0.5 mM	1 mM
Wild type	88.8 ± 5.4	80.2 ± 3.6	2.7 ± 4.6
GFP	90.0 ± 3.3	73.1 ± 15.7	1.3 ± 1.1
LmjMCA–GFP	91.4 ± 1.9	0.0	0.6 ± 1.0
ΔN-LmjMCA–GFP	81.3 ± 3.1	1.1 ± 1.9	1.1 ± 1.9
cd–GFP	83.9 ± 7.1	2.2 ± 1.9	2.7 ± 4.6
N63–GFP	98.7 ± 2.3	92.3 ± 4.6	1.5 ± 1.3
LmjMCA-FLAG	93.6 ± 3.9	3.0 ± 0.9	0.4 ± 0.7
LmjMCA-FLAG H147A/C202A	89.3 ± 3.2	94.1 ± 4.1	1.8 ± 3.2

**Fig. 7:**
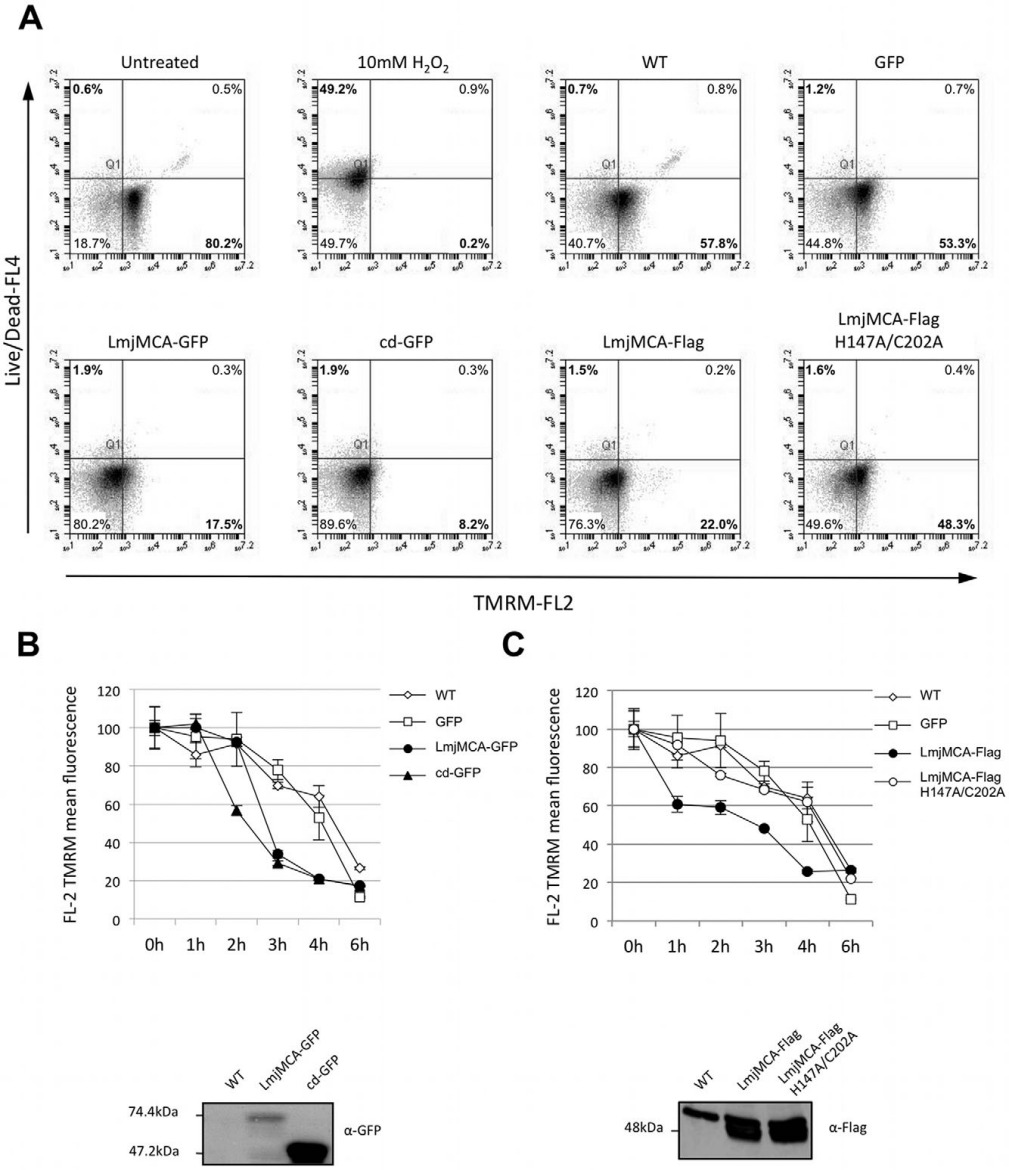
Mitochondrial membrane potential. Parasites were exposed to 0.5 mM H_2_O_2_, harvested every hour, incubated with the TMRM dye and analysed by flow cytometry to detect the loss of mitochondrial membrane potential. A. Mitochondrial membrane potential and cell integrity analysis after 3 h H_2_O_2_ treatment of parasites expressing wild type (WT), GFP, LmjMCA–GFP, cd–GFP, LmjMCA-Flag and LmjMCA-Flag H147A/C202A. Negative (untreated) and positive (10 mM H_2_O_2_, 1 h) controls are shown in the two first dot plots respectively. Percentages of healthy (TMRM-positive, Live/Dead-negative) and necrotic (TMRM-negative, Live/Dead-positive) cells are shown in bold. B. Mitochondrial membrane potential analysis over 6 h H_2_O_2_ treatment of parasites expressing WT, GFP, GFP-tagged full-length metacaspase (LmjMCA–GFP) and metacaspase catalytic domain (cd–GFP). C. Mitochondrial membrane potential analysis over 6 h H_2_O_2_ treatment of parasites expressing WT, GFP, Flag-tagged full-length metacaspase (LmjMCA-Flag) and its catalytically inactive version (LmjMCA-Flag H147A/C202A). Lower panels show immunoblots of protein extracts from each analysed sample with the level of expression of LmjMCA–GFP, cd–GFP and LmjMCA-Flag, LmjMCA-Flag H147A/C202A using anti-GFP and anti-Flag antibodies respectively.

In order to quantify this mitochondrial phenotype and to link it to an apoptotic-like process, we analysed the loss of mitochondrial membrane potential using the specific sensor TMRM. As evidenced in Fig. 7A, after 3 h of H_2_O_2_ treatment at 0.5 mM, wild-type parasites or parasites expressing GFP only showed over 50% of viable cells with unaffected mitochondrial membrane potential whereas, in parasites overexpressing LmjMCA–GFP or the catalytic domain cd–GFP, the percentage of cells with unaffected mitochondrion decreased below 20%. The same effect was observed with parasites overexpressing LmjMCA-Flag in comparison with wild-type parasite or parasites expressing the catalytically inactive form of MCA. At the same time, the number of necrotic cells (Live/Dead positives) did not increase over 2% suggesting that metacaspase is indeed participating in an apoptotic-like process.

We further analysed this phenotype at different time points. In LmjMCA–GFP-expressing parasites, the 0.5 mM H_2_O_2_ treatment provoked a mitochondrial depolarization after 2 h whereas parasites overexpressing cd–GFP strongly decreased their mitochondrial membrane potential already after 1 h (Fig. 7B). This strong effect could be due to the high expression level of cd–GFP (Fig. 7B, lower panel). LmjMCA-Flag-expressing parasites also exhibited mitochondrial depolarization at 1 h of treatment while the catalytically mutated form did not (Fig. 7C). After 6 h of treatment, we could no longer observe differences between the different lines. Interestingly, the percentage of necrotic cells as measured with the Live/Dead reagent did not increase over 2% even after 6 h of H_2_O_2_ treatment (data not shown).

## Discussion

Metacaspases are cysteine proteases with a caspase-like domain expressed in plants, yeast and protozoan parasites. As shown for plants and *Leishmania* protozoan parasites, their substrate specificity differs from caspases since they recognize preferentially basic amino acids, mainly arginine, in the P1 position. The function of MCA in cell death has been shown in plants and in yeast depending on the type of inducer used. MCA is autoprocessed and when expressed in yeast cells, the enzymatic activity of the catalytic domain of *L. major* MCA was shown to be essential in complementing yeast metacaspase function in the cell death pathway (González *et al*., 2007). Although there is evidence for MCA autoprocessing and maturation of an active catalytic domain from a primary translation product, the processing sites have not been determined for any of the MCA in *Leishmania*. LmjMCA is an arginine cysteine protease, which likely needs to be processed to reach higher levels of activity as demonstrated in yeast (González *et al*., 2007) and, in *Leishmania*, its overexpression was shown to participate in cell death (Lee *et al*., 2007). Similarly to other MCAs, thus far, there was no evidence of its processing and the role of LmjMCA-cd in *Leishmania* cell death.

In higher eukaryotes, the activation of caspases depends on the cleavage of the p20 and p10 subunits either by themselves (initiator caspases) or by other caspases (effector caspases) and on their interaction. In this study, cleavage products of LmjMCA in *L. major* parasites were detected. However, overexpressing only the catalytic domain was sufficient in yeast to promote cell death, and our present results in *Leishmania* suggest that the enzymatically active LmjMCA does not depend on the interaction of two heterologous subunits as in higher eukaryote caspases and confirmed that this domain is sufficient to act in cell death.

In this study, we determined the cellular localization of LmjMCA and the possible sites that are important for the maturation of LmjMCA catalytic domain in physiological and in stress situations. Unfortunately, the low level of expression of LmjMCA precluded purification of the processed fragment and identification by N-terminal sequencing. Furthermore, there is no LmjMCA null mutant available, which means that the described experiments have been performed with parasites harbouring an active endogenous metacaspase. Despite these technical constraints, we can conclude that in *L. major*, MCA is not fully processed and the catalytic domain is present on different LmjMCA polypeptides depending on the cleavage at the N-terminus or at the C-terminus. Three polypeptides containing mainly the catalytic domain have been identified (26, 24 and 18 kDa). Based on experimental data and sequence comparison between species and preliminary mutagenesis studies, we propose that they are the products of processing at R63 and R298 for the 26 kDa, R75/79 and R289 for the 24 kDa and R135 and R298 for the 18 kDa polypeptide. Our preliminary *in vitro* mutagenesis data allow us to exclude cleavage at position R75 and confirmed N-terminal cleavages at positions R79 and R298. Although substrate specificity of metacaspases is mainly directed towards arginine in P1 position, we cannot exclude cleavages at lysine. According to the positions of arginines and of lysines in the LmjMCA sequence and the molecular masses detected, no cleavage at lysine could be accounted for the N-terminus of the LmjMCA. In contrast, at the C-terminal limit of the catalytic domain, lysine upstream of R298 (i.e. K295 and K296) are conserved among all *Leishmania* species but do not appear to act alternatively to R298. Although the positions of the cleavage sites await further confirmation, we are confident that the N- and C-terminus of the catalytic domain are positioned in these regions, and likely limited by R63 or R79 and R135 at the N-terminus and most likely R298 at the C-terminus. *Leishmania* metacaspase sequences were also compared with TbMCA2, the *T. brucei* metacaspase LmjMCA homologue. Although the previously described cleavage sites of TbMCA2 (Moss *et al*., 2007) were not conserved in *Leishmania*, most of the cleavage sites that we identified in *Leishmania* are present in TbMCA2 in which an arginine R74 is aligned with LmjMCA R63 and lysines K101 and K155 are aligned with LmjMCA R86 and R135, respectively, suggestive of a common origin.

According to the functional heterologous complementation of a *S. cerevisae* metacaspase null mutant by a polypeptide encoding *LmjMCA* catalytic domain (AA 63–314), we could hypothesize that the catalytic domain could be sufficient to induce cell death in *Leishmania*. We tested this hypothesis and showed that a cytoplasmic *Leishmania* catalytic domain (AA 117–298) would enhance sensitivity of parasites to ROS such as H_2_O_2_. This function was dependent on the catalytic activity and not on any other property of this domain since mutating the catalytic dyad of the active site abrogated this increased sensitivity. Interestingly, this polypeptide is mainly found in the NP-40-insoluble fraction in physiological situations, but in the soluble fraction in the dying parasites. The reasons for such a translocation into an insoluble fraction is unknown but we could postulate that this difference in solubility could be correlated with a release of LmjMCA catalytic domain out of the mitochondrial compartment or more likely from a release of the catalytic domain out of interacting proteins which are degraded or modified upon cell death induction. In terms of regulating the cell death pathway in *Leishmania*, this observation could be quite relevant. When parasites are exposed to HS, H_2_O_2_, LmjMCA primary translation products and most of the LmjMCA precursors are degraded. A similar result was obtained with anti-*Leishmania* drugs such as miltefosine or curcumin; interestingly it has recently been proposed that miltefosine induces PCD in *L. donovani* through mitochondrial dysfunction and cytochrome *c* release (Verma *et al*., 2007).

In a previous study, we proposed that the release of lysosomal enzymes is a possible mechanism implicated in *Leishmania* death (Zangger *et al*., 2002). Rupture of the lysosomal membrane would release cathepsins which are highly abundant in *Leishmania* and which could cleave LmjMCA and interacting partners. The importance of other proteases in the cell death process has been described in other organisms. In yeast cells transfected with the inactive complete sequence of LmjMCA, a product of the N-terminal cleavage of LmjMCA was also found (González *et al*., 2007). These results suggest that the N-terminal domain of LmjMCA is cleaved independently of its autoprocessing activity. Similarly, the cleavage of the N-terminal domain of the *Arabidopsis thaliana* metacaspase AtMC9 by an upstream protease seemed to be a prerequisite for the induction of its autoprocessing and activity (Belenghi *et al*., 2007).

The LmjMCA N-terminal domain could encode an MLS, which we previously demonstrated to localize LmjMCA to the mitochondrion (Ambit *et al*., 2008). Furthermore, LmjMCA was also localized to the kinetoplast and the nuclear mitotic spindle in mitotic parasites (Ambit *et al*., 2008). In the present investigation, antibodies recognizing the catalytic domain of LmjMCA and GFP-tagged LmjMCA allowed the localization of the protein mainly in the cytoplasmic compartment but some LmjMCA was also present in the mitochondrion. No LmjMCA was detected on the mitotic spindle, in contrast to our previous study with a different anti-LmjMCA catalytic domain antibody. The retention in the cytoplasm could be due to the presence of a sequence (AA 64–101) containing hydrophobic residues. Such a sequence could interfere with the mitochondrion translocation machinery by hiding the MLS and thus affecting translocation efficiency as can be deduced from experiments on the cellular localization of N101–GFP, which failed to be targeted to the mitochondrion. This controlled targeting could be important in the delivery of limited amount of specific LmjMCA polypeptides into the mitochondrial compartment.

Knowing that MLS is required for translocation, our results indicate that some LmjMCA precursors are translocated into the mitochondrion and both N- and C-terminal cleavages are used in the maturation of the catalytic domain. It also suggests that the enzymatic activity of LmjMCA is present in the cytoplasm and in the mitochondrion. Such a dual repartition has been reported for caspases. For example, caspase-9 can be found in the mitochondria of certain tissues and is implicated in the release of cytochrome *c* (Scaffidi *et al*., 1999). Similarly, in yeast, PCD-induced cytochrome *c* release has been shown to be metacaspase dependant (Guaragnella *et al*., 2010). Caspases-2 and -3 have also been described to be present in the cytosol and associated with the inner mitochondrial membrane (Mancini *et al*., 1998; Samali *et al*., 1998) and each caspase pool was shown to undergo differential processing according to the apoptotic signal (Yuan *et al*., 2001).

In conclusion, we provide experimental evidence of the functional role of the catalytic domain of LmjMCA in the cell death pathway of *Leishmania* parasites. When cell death is induced by an oxidative stress or by anti-*Leishmania* drugs, LmjMCA is processed and the catalytic domain is released. From our previous results in yeast and the results of the present study, it is likely that the released catalytic domain is enzymatically active and could be directly involved in the proteolytic degradation occurring in dying parasites. Whether LmjMCA catalytic domain acts directly or indirectly on the mitochondrion and whether the dual localization of LmjMCA and its catalytic domain are important in the mitochondrial loss of function require further investigations. In any case, our results provide evidence of a pathway induced when parasites are driven to death. Such a pathway could be conserved in other protozoan parasites encoding metacaspase and its activation could be relevant for the design of new anti-protozoan therapeutics.

## Experimental procedures

### Metacaspase sequence analysis

The metacaspase primary amino acid sequences from different *Leishmania* species were obtained from Uniprot online database. The following sequences were used: *L. major* (Q4FWW2), *L. donovani* (Q0ZL29), *L. mexicana* (B6DU81), *L. amazonensis* (B6DU80) as representatives of *Leishmania Leishmania* subgenus, *L. tarentolae* (B6DU79) as a *Sauro-Leishmania* representative and *L. braziliensis* (B6DU82) and *L. guyanensis* (B6DU83) as representatives of *L.* (*Viannia*) subgenus. Sequences were aligned using clustal w2 multiple sequence alignment program.

### Chemicals

Restriction enzymes were purchased from New England Biolabs (Ipswich, MA) and reagents, unless otherwise stated, were purchased from Sigma-Aldrich (St. Louis, MO).

### Molecular constructs

The complete nucleotide sequence of *LmjMCA* was first amplified with primers N-term_HindIII_fwd (5′-CCC AAG CTT GGG ATG GTG CAG GTG CCG CAG-3′) and C-term_PacI_rev (5′-CCT TAA AGC CAG GCG GGA GTG GGC T-3′). PCR products were digested with HindIII and PacI restriction enzymes and inserted into the pKT128 yeast cloning vector which expresses a gene coding for GFP (yEGFP) that has been optimized to give better fluorescence (Sheff and Thorn, 2004). *LmjMCA–GFP* gene was then amplified with primers MCA-gfpEcoRI_fwd (5′-GGC GGA TTC ATG GCA GAC CTT TTT GAT A-3′) and MCA-gfpEcoRI_rev (5′-GGC GAA TTC CCT TAT TTG TAC AAT TCA TCC-3′). The PCR product was digested with EcoRI and inserted into the pNUS–GFPcN vector (Tetaud *et al*., 2002) to generate the fusion protein LmjMCA–GFP (74.4 kDa).

The DNA sequences encoding the 63 and 101 first amino acids of LmjMCA were amplified using the primers MCompGFP Fw (5′-CGC CAT ATG GCA GAC CTT TTT GAT AT-3′), N63-BglII-rev_2 (5′-GCG AGA TCT ACG GAC ACG GAC ACG GCC GCC-3′) and MCompGFP Fw (5′-CGC CAT ATG GCA GAC CTT TTT GAT AT-3′), Nterm_101aa_bglII (5′-CGC AGA TCT CTC GCT AAT CGG GAA-3′), respectively, and inserted into the pNUS–GFPcN plasmid using NdeI and BglII restriction sites to generate the fusion proteins N63–GFP (34.2 kDa) and N101–GFP (38.2 kDa). The pNUS–GFPcN vector expressing GFP (27 kDa) was used as a negative control. The DNA-encoding regions corresponding to the catalytic domain (AA 117–298) and LmjMCA lacking the N-terminal domain (AA 117–435) were amplified using the following primers ΔN-fwd-bglII (5′-CCA TAT GAG ATC TCC TTC CCG ATT AGC-3′), MCctrGFP Rv (5′-GCG AGA TCT ACC TTT TTT GCG CAG-3′) and ΔN-fwd-bglII (5′-CCA TAT GAG ATC TCC TTC CCG ATT AGC-3′), DNC–GFP Rv (5′-CGC AGA TCT ACC TTT TTT GCG CAG-3′) respectively and inserted into the BglII restriction site of pNUS–GFPcN plasmid to encode the fusion proteins cd–GFP (47.2 kDa) and ΔN-LmjMCA–GFP (62.03 kDa). *LmjMCA-Flag* was amplified from the pESC-LCA plasmid (González *et al*., 2007) with the primers MCA-FWD-EcoRI (5′-GCG GAA TTC ATG GCA GAC CTT TTT G-3′) and MCA-REV-EcoRI (5′-GCG GAA TTC GCC AGG CGG GAG TG-3′) and inserted into the pNUS–GFPcN vector at the EcoRI restriction sites. Point mutations in the *LmjMCA–GFP* and *LmjMCA–Flag* coding gene were introduced with the Quick Change® II XL Site-directed mutagenesis kit (Stratagene).

### Parasite cultivation and transfection

*Leishmania major* wild-type MRHO/IR/75 promastigotes were grown in M199 medium (Invitrogen AG, Basel, Switzerland) complemented with 100 U ml^−1^ penicillin, 100 µg ml^−1^ streptomycin, 10% heat-inactivated Fetal Calf Serum (FCS – Seromed GmbH, Wien, Switzerland), 40 mM *N*-2-hydroxyethylpiperazine-*N′*-2-ethane-sulphonic acid (HEPES) and 0.1 mM adenine, until stationary phase was reached. One hundred thousand parasites were harvested, washed once in four volumes of Electroporation Buffer (EPB: 21 mM HEPES pH 7.4, 137 mM NaCl, 5 mM KCl, 0.7 mM Na_2_HPO_4_, 6 mM glucose) diluted to 4 × 10^7^ parasites in 400 µl of EPB, transfected with 40 µg of DNA by electroporation using the Bio-Rad Gene Pulser Unit at 0.45 kV and capacitance of 500 µF. Parasites were then transferred to 10 ml of the same culture medium, incubated overnight at 25°C, centrifuged, resuspended in 0.5 ml of culture medium, and spread onto freshly prepared M199 plates (M199 medium, 10% FCS, 40 mM HEPES, 0.1 mM adenine, 4.2 mM NaHCO_3_, 2% BactoAgar and 16 µg ml^−1^ geneticin).

### Cell death induction

To induce cell death, parasites were incubated with 0.5 or 1 mM H_2_O_2_ during 1 h (Fig. 4) or 4 h (Fig. 5) in complete culture medium or heat shocked at 55°C during 10 min then incubated for 1 h at 26°C. Drug-induced cell death was obtained by cultivating logarithmic-phase parasites in the presence of 40 µM miltefosine or 100 µM curcumin in complete M199 culture medium for 10 h at 26°C.

### Parasite cell lysis and protein quantification

Soluble and insoluble protein fractions from *L. major* were obtained after lysis in a solution containing 0.14 M NaCl, 1.5 mM MgCl_2_, 10 mM Tris-HCl, 0.5% Nonidet P-40 (NP-40), 2 mM EDTA and a cocktail of protease inhibitors composed of 40 ng ml^−1^ leupeptin, 10 µg ml^−1^ pepstatin, 0.8 mM OPA (*ortho*-phenanthroline) and 160 µM E64 [*trans*-epoxysuccinyl-leucylamido (4-guanidino) – butane], incubated for 1 min on ice then centrifuged for 5 min at 4°C to separate the supernatant (soluble proteins) from the pellet (insoluble proteins). Cell fractionation in the presence of digitonin was performed according to Foucher *et al*. (2006) and A. Schneider (pers. comm.). Briefly, 2 × 10^8^ parasites were washed in 1× wash buffer (20 mM Tris-HCl pH 7.9, 20 mM glucose, 0.15 M NaCl), the pellet resuspended in 750 µl of digitonin/SoTE 100 µM solution (SoTE: 20 mM Tris-HCl, 0.6 M sorbitol, 2 mM EDTA, pH 7.5) containing a cocktail of protease inhibitors, then incubated for 5 min on ice. A volume of 250 µl was saved as total proteins fraction. One hundred and fifty microlitres of 0.3 M sucrose was added to maintain cell structures during the centrifugation (5 min, 13 000 r.p.m. at 4°C). The supernatant was saved as fraction C (cytosolic proteins). To obtain fraction M (mitochondrial proteins), the pellet was resuspended in 500 µl of 500 µM digitonin/SoTE solution and the same protocol was repeated. Protein fractions were stored at −70°C until use. Protein concentration was quantified using a BCA protein assay reagent (Pierce Biotechnology, Rockford, IL) with bovine serum albumin (BSA) as standard.

### Immunodetection

Twenty micrograms of protein samples were separated by SDS-PAGE. Low-range molecular weight standards were used (Bio-Rad Laboratories, Hercules, CA). Proteins were then transferred to a nitrocellulose membrane by electroblotting and incubated either with the rabbit polyclonal RE53 anti-LmjMCA antibodies (produced against a synthetic 76-amino-acid peptide E53 comprising the caspase-like domain of the *T. brucei* metacaspase) (González *et al*., 2007), with the mouse monoclonal anti-GFP antibody (Roche Diagnostics AG, Switzerland) or with the mouse monoclonal anti-Flag M2 antibody. Digitonin fractionation was controlled using specific antibodies: anti-mTPXN (mitochondrial tryparedoxin peroxydase) (Castro *et al*., 2002), anti-cTPXN (cytoplasmic tryparedoxin peroxydase) (Acestor *et al*., 2006) and anti-cytochrome *c* (H-104 from Santa Cruz Biotechnology, Santa Cruz, CA). Membranes were then incubated with the corresponding secondary antibody coupled to horseradish peroxidase (Promega Corp., Madison, WI) and developed by enhanced chemiluminescent staining using ECL™ Western blotting system (Amersham Biosciences, Piscataway, NJ).

### Test for apoptotic marker

One million logarithmic-phase parasites were treated with 1 mM H_2_O_2_ in 1 ml of M199 complete medium for 4 h, washed and resuspended in 1× annexin V binding buffer (10 mM HEPES/NaOH, pH 7.4, 140 mM NaCl, 2.5 mM CaCl_2_, 1.2 M sorbitol) and incubated for 15 min at room temperature with 10 µl of annexin V-FITC (Invitrogen AG) and 10 µl of 500 ng ml^−1^ PI. Ten thousand events were accumulated with a BD FACScan apparatus and data were analysed using the CellQuest™ (Becton Dickinson Biosciences, San Jose, CA) and Flowjo™ (Tree Star, Ashland, OR) softwares.

### Confocal microscopy

*Leishmania major* live promatigotes expressing GFP-tagged proteins were treated during 15–30 min with 100 nM red Mitotracker® (Invitrogen) and fixed in 4% freshly made formaldehyde then immobilized by incubation on poly-l-lysine-coated optic glass bottom plates (NUNC, Roskilde, Denmark) for at least 30 min. For immunofluorescence, wild-type parasites were permeabilized with 0.1% Triton X-100 in 1× PBS (phosphate-buffered saline) for 10 min, incubated in a blocking solution containing 0.1% Triton X-100 and 1% BSA in 1× PBS for 1 h then incubated with the primary antibody (rabbit anti-LmjMCA diluted in 0.1% Triton X-100 and 0.1% BSA in 1× PBS) for 2 h at room temperature followed by 1 h incubation with the secondary antibody (anti-rabbit IgG-Alexa 488 diluted in 0.1% Triton X-100 and 0.1% BSA in 1× PBS). DAPI staining was performed during 10 min at room temperature with a dilution of 1/500 from a stock at 10 µg ml^−1^ in 1× PBS.

Fluorescent imaging was performed with an Inverted Zeiss LSM 510 Meta confocal microscope (Carl Zeiss). GFP and Mitotracker were excited with 488 nm argon multiline 30 mW and 543 nm HeNe 1 mW lasers respectively. Image acquisition was performed with an AxioCam MRm camera using a Plan-APOCHROMAT 63×/1.40 or Plan-NEOFLUAR 40×/1.30 oilimmersion objective for Fig. 1A. Images were processed using the LSM Software Rel. 3.5. At least 100 parasites treated with H_2_O_2_ (0, 0.5 and 1 mM) and stained with Mitotracker were counted and the percentage of cells with intact mitochondrion was determined for each condition. Means and standard deviations from three separate experiments were calculated.

### Loss of mitochondrial membrane potential

Logarithmic-phase promastigotes were incubated with 0.5 mM H_2_O_2_. Cells were harvested every hour, cell death induction was stopped with 250 U ml^−1^ catalase and then cells were incubated with 50 nM tetramethylrhodamin methyl esther perchlorate (TMRM, Sigma CAT #T5428) for 30 min and analysed by C6 ACCURI® flow cytometer. Fluorescence was detected in FL2. The experiment was performed in triplicates, normalized to 100% for untreated parasites. Standard deviation bars are shown for each time point.

To test viability, parasites were stained in parallel with the LIVE/DEAD Assay (Invitrogen, CAT #L10120) using a 1:1000 dilution. Fluorescence was detected in FL4.
