# Supplementary material for: Processing of metacaspase into a cytoplasmic catalytic domain mediating cell death in Leishmania major
Source: Mol Microbiol. 2011 Jan;79(1):222–39. doi: 10.1111/j.1365-2958.2010.07443.x (PMC3047009; doi:10.1111/j.1365-2958.2010.07443.x)
Supplement: Supplementary file 1 [file mmi0079-0222-SD1.pdf]

Figure S1

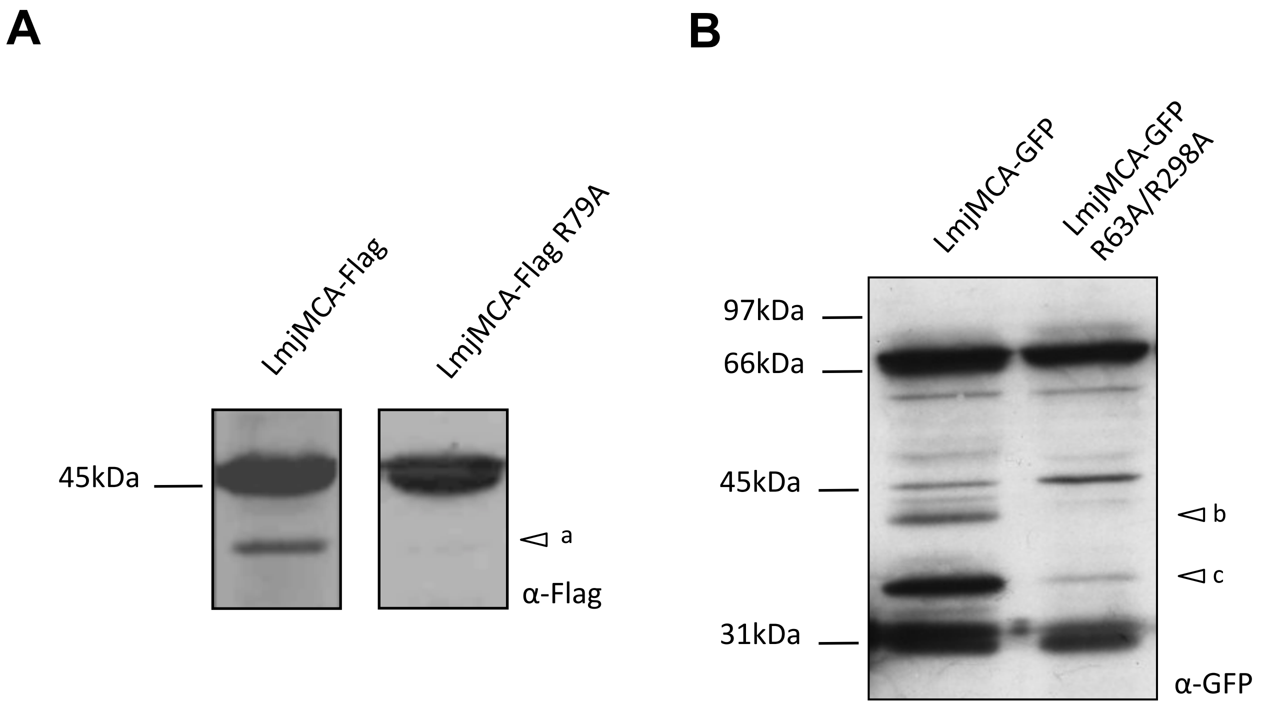

Fig. S1. Mutagenesis of N- and C-terminal cleavage sites of LmjMCA. Amino acid R79 of LmjMCA-Flag (A) and amino acids R63 and R298 of LmjMCA-GFP (B) were changed to alanines and processing profiles were compared to non mutated LmjMCA by immunoblot using anti-Flag and anti-GFP antibodies respectively. Arrowheads a and b show metacaspase processing fragments migrating at the expected molecular weight of 39 kDa after the processing of LmjMCA-Flag at R79 and 42 kDa after the processing of LmjMCA-GFP at R298 respectively. Arrowhead c shows a processing fragment of LmjMCA-GFP migrating at an approximate molecular weight of 34 kDa.
